# Rapid Fermentable Substance Modulates Interactions between Ruminal Commensals and Toll-Like Receptors in Promotion of Immune Tolerance of Goat Rumen

**DOI:** 10.3389/fmicb.2016.01812

**Published:** 2016-11-17

**Authors:** Hong Shen, Zhongyan Lu, Zhan Chen, Yufeng Wu, Zanming Shen

**Affiliations:** ^1^College of Life Science, Nanjing Agricultural UniversityNanjing, China; ^2^Bioinformatics Center, Nanjing Agricultural UniversityNanjing, China; ^3^Key Lab of Animal Physiology and Biochemistry, College of Veterinary Medicine, Nanjing Agricultural UniversityNanjing, China; ^4^College of Agriculture, Nanjing Agricultural UniversityNanjing, China

**Keywords:** rumen microbiota, toll-like receptors, immune tolerance, dietary modulation, epithelium transport, microbe–host interactions

## Abstract

Whether dietary non-fiber carbohydrate (NFC), a rapid fermentable substance, affects immune homeostasis of rumen through the modulation of interactions of ruminal microbiota and epithelial toll-like receptors (*TLRs*) remains unclear. A combination of 16S rRNA amplicon sequencing and quantitative PCRs was applied to study the synergetic responses of ruminal microbiota and epithelial *TLRs* to the dietary NFC switch from 15 to 31% in the goat model. The results showed that the 31% NFC diet caused the radical increases on the richness and diversity of rumen microbiota. The phylum Verrucomicrobia was most significantly expanded, whereas opportunistic pathogens, namely *Rikenella*, *Anaeroplasma*, and *Olsenella*, were significantly decreased. In rumen epithelium, the significantly increased expressions of *TLR1*, *6*, *10* were associated with the significantly decreased expressions of pro-inflammatory cytokines interleukin-1beta (*IL-1ß*), *IL-6*, and anti-inflammatory cytokine *IL-10*. Constrained correlation analysis indicated that the increased abundance of commensal bacteria in Verrucomicrobia subdivision 5 contributed to the upregulation of *TLR10* expression. Finally, the significantly increased concentrations of rumen short-chain fatty acids (SCFAs), coupled with the significantly upregulated expressions of epithelial genes related to SCFA absorption were observed in goats fed with 31% NFC diet. Thus, the NFC-induced expansion of rumen microbiota promoted epithelium tolerance by enhancement of the intensity of *TLR10* signaling. The newly established equilibrium benefited to the transport of ruminal energy substances into the blood.

## Introduction

The rumen is the most important site for digestion and absorption in ruminant animals. Rumen digestion is performed by symbiotic microbes, and the major fermentation productions, short-chain fatty acids (SCFAs), absorbed by the rumen epithelium, meet 50–70% energy requirement of the animals. However, persistent contact of the rumen epithelium with trillions of bacteria brings serious threatens to the host healthy. To maintain a harmonious symbiosis with these exogenous antigens, the host has to be tolerant of commensals. Tolerance is defined as a state in which the immune system of the host rendered non-reactive toward commensals (van Baarlen et al., 2009). The commensals in the tolerance state contribute to the energy absorption of the host by modulating the expression of host genes that participate in breakdown and absorption of energy substrates/diverse and fundamental physiological functions (Hooper and Gordon, 2001). The acquiring of tolerance needs the activation of innate and adaptive responses in the prevention of an inappropriate inflammatory response (Wood et al., 2012; Belkaid and Hand, 2014; Swiatczak and Cohen, 2015). In animals, the gastrointestinal (GI) microbiota has a crucial function in building and maintaining gut tolerance. A dysbiosis of GI microbiota predisposes the host to various kinds of immune and metabolic diseases (Ohland and Jobin, 2015).

Previous studies have shown that diet contribute to the immune homeostasis by promoting the SCFAs productions of GI microbiota, which modulate the immune responses via G protein-coupled receptors (GPRs) and histone deacetylases (HDACs) pathways (Tan et al., 2014). A study on mice revealed that diet enhanced immune tolerance also by affecting the interactions of pattern-recognition receptors (PRPs) and commensals in GI tract (Zelante et al., 2013). Our previous studies have shown that the increased intake of non-fiber carbohydrate (NFC), which can be rapidly fermented by GI microbes, causes the significant increases in the concentrations of butyrate, acetate and propionate in goat rumen (Yan et al., 2014; Lu et al., 2015). However, yet little is known about the diet-induced specification of ruminal microbiota and their effects on the immune tolerance of rumen epithelium.

Toll-like receptors (*TLRs*) have a critical role in suppressing inflammation of the GI epithelium. Mice exhibits an increased susceptibility to intestinal inflammation when they are deficient in myeloid differentiation primary response 88 (*MyD88*), an important component of the *TLR* signaling cascade (Frantz et al., 2012; Chu and Mazmanian, 2013). *TLRs* are also reported to suppress the inflammatory responses by reducing inflammatory cytokine productions (Oosting et al., 2014), by conditioning tolerance CD103^+^ dendritic cells (DCs) (DePaolo et al., 2012), and by modulating the development of regulatory T cells (Tregs) (Kramer et al., 1996). Although, no similar report concerning the functions of *TLRs* in maintaining the rumen homeostasis is presently available, the study of Malmuthuge et al. (2012) has revealed that *TLR 1–10* is constantly expressed in ruminal epithelium. We therefore speculate that the interactions between ruminal microbiota and epithelial *TLRs* play important roles in promotion of immune tolerance during dietary modulation.

In this study, a combination of molecular microbiology and immunology methods has been applied to investigate the synergetic responses of the rumen microbiota and the expressions of *TLRs* at the apical surface to the switch of dietary NFC from 15 to 31%. The expressions of SCFA-absorption-related genes were investigated to understand the effects of altered microbiota on the epithelium functions. These results provide a better understanding of the nature of the host–microbe interactions.

## Materials and Methods

The study was approved by the Animal Care and Use Committee of Nanjing Agricultural University, in compliance with the Regulations for the Administration of Affairs Concerning Experimental Animals (The State Science and Technology Commission of P. R. China, 1988).

### Animals

Six male goats (Boer × Yangtze River Delta White, aged 4 months, 14–18 kg of bodyweight) 14–18 kg were purchased from local farm. Before, the feeding experiment, all goats were kept together in an open air yard and fed a pure hay diet ad libitum for 14 days to adapt the new environment. After the adaptation period, all the goats received a LNFC diet consisting of 90% hay plus 10% concentrate (15% NFC) in the first 4 weeks. Subsequently, the goats were randomly assigned into two groups. One group of three goats (referred to as the LNFC group) was slaughtered to collect the ruminal fluid and epithelium on day 28. The remaining group of three goats (referred to as the MNFC group) received a MNFC diet consisting of 65% hay plus 35% concentrate (31% NFC) in the following 4 weeks. The ruminal fluid and epithelium of MNFC group were collected on day 56. During the feeding experiment, all the goats were placed in individually pens (1.2 m × 1.0 m) and fed in two equal portions of designed diet at 0800 and 1700 h daily. The composition of the MNFC and LNFC diets is presented in Supplementary Table S1. Water was freely available to all goats during the experiment.

### Sample Collection

Ruminal fluid samples were taken just before matinal feeding (0 h) and at 1.5, 3, 4.5, and 6 h after matinal feeding on day 28 in the LNFC group and on day 56 in the MNFC group. An aliquot (20 mL) of ruminal fluid was strained through the four-layer cheesecloth and immediately subjected to pH measurement. Thereafter, a 5% HgCl_2_ solution (1 mL) was added, and the sample was stored at -20°C for the determination of the SCFA concentration. Goats were slaughtered at 8 h after matinal feeding on day 28 in the LNFC group and on day 56 in the MNFC group. Immediately after slaughter, approximately 5 mL ruminal fluid was collected for microbiota analysis. Rumen tissue from the ventral blind sac was quickly excised and washed repeatedly in ice-cold phosphate-buffered saline (PBS; pH 7.4) until the PBS was clear. The epithelium was separated from the muscle layers and stored at -80°C until RNA extraction.

The ruminal SCFA concentration was determined by using a chromatograph (HP6890N, Agilent Technologies, Wilmington, DE, USA) as described by Yang et al. (2012).

### Ruminal Microbiota Analysis

The metagenomic DNA of the microbiota was extracted from the ruminal fluid by using a Bacterial DNA Kit (Omega). The DNA concentration was determined in a Nanodrop 1000 (Thermo Fisher Scientific, Wilmington, DE, USA) and stored at -20°C until further processing. The 16S rRNA amplicon library preparation was performed by PCR amplification of the V3–V4 region of the 16S rRNA gene with the universal primers 319F (5′-ACTCCTACGGGAGGCAGCAG-3′) and 806R (5′-GGACTACHVGGGTWTCTAAT-3′) (Mori et al., 2014), including TruSeq adapter sequences and indices. All libraries were sequenced on an Illumina MiSeq platform (Illumina, San Diego, CA, USA) with the paired-end chemistry (PE300).

Paired reads were filtered for quality (Q30) and joined by using FLASH version 1.2.11 (Magoc and Salzberg, 2011). Sequences that contained read lengths shorter than 400 bp were removed and classified into taxa by blasting with the ribosomal database project (RDP) database at a 97% similarity threshold. Operational taxonomic units (OTUs) were hierarchically summed at all taxonomic levels, and the counts were normalized to relative abundance for each sample. The richness and diversity of the microbial communities was estimated by using the R program phyloseq package (McMurdie and Holmes, 2013). For a deeper analysis of the diversity of the major evolutional clades in the ruminal microbiota, all data were filtered to require a relative abundance of at least 1% in at least one sample. Then, MUSCLE version 3.8.31 (Edgar, 2004) was employed to align the complete 16S rRNA sequences of the corresponding species in the RDP database, and RAxML version 8 (Stamatakis, 2014) was used to construct the phylogenetic tree. The tree was plotted by means of the R program ape package (Paradis et al., 2004).

Significantly different OTUs between the groups were identified by using a linear discriminant analysis (LDA) with LEfSe (Segata et al., 2011). The relationships between the abundance of each biomarker genera and the expression of the host genes were explored by constrained correspondence analysis (CCA) of the vegan package (Oksanen et al., 2016). The genes used in the CCA analysis were significantly different as shown by the *t*-test (*p* < 0.05). The R program ggplot2 package (Wickham, 2009) was employed to generate the visual interpretation (biplot) of the gene-microbiota relationships. The coordinates of the arrows on the plot were determined by using the expression of the genes, and the coordinates of the points were determined by using the relative abundance of the genera.

### Quantitative PCR

Total RNA was extracted from the ruminal epithelium by using the RNeasy Mini Kit (Qiagen, Shanghai, China). A random hexamer primer (Invitrogen, Shanghai, China) and moloney murine leukemia virus (M-MLV) reverse transcriptase (Fermentas, Burlington, ON, Canada) were employed to synthesize the cDNA. Quantitative PCR was performed by using the StepOne Plus real-time PCR system (Applied Biosystems, Den Ijssel, Netherlands) and SYBR-Green (Roche, Shanghai, China) for detection. Glyceraldehyde-3-phosphate dehydrogenase (GADPH) was chosen as the housekeeping gene. The primers of 11 genes were designed in this study according to the available sequences in NCBI, and the primers of the remaining 11 genes were synthesized according to the description of Yan et al. (2014) (Supplementary Table S2). The amplification efficiency of the primers was determined by means of a dilution series of epithelial cDNA. All samples were run in triplicate, and the data were analyzed according to the 2^-ΔΔCT^ method (Livak and Schmittgen, 2001). The identity and purity of the amplified product were checked by analysis of the melting curve obtained at the end of the amplification.

### Database Submission

The sequencing data are available in the NCBI under BioProject PRJNA305843.

## Results

### Structure of Commensal Communities

**FIGURE 1:**
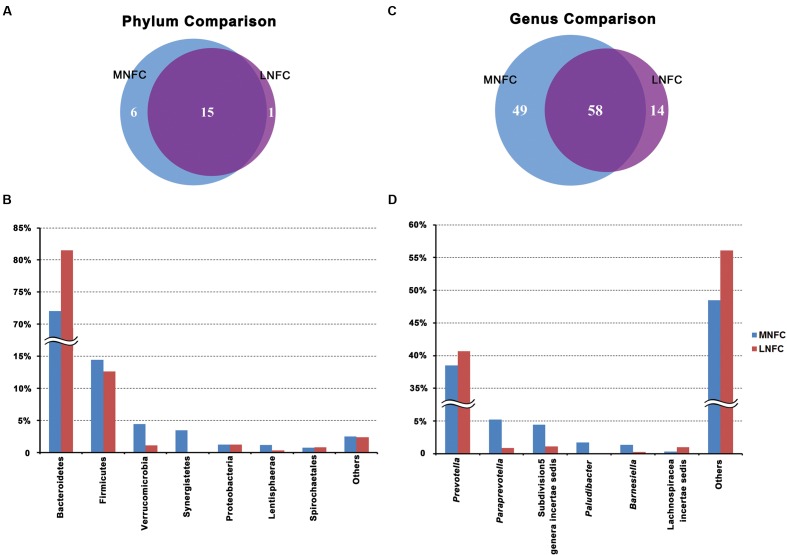
**(A)** Venn diagram showing the overlap of phyla between groups. **(B)** Phylum-level comparison of bacterial operational taxonomic units (OTUs)between the groups. **(C)** Venn diagram showing the overlap of genera between groups. **(D)** Genus-level comparison of bacterial OTUs in the BT group.

At the phylum level, a total of 22 prokaryotic phyla were identified by comparing with the RDP databases at a 97% similarity threshold, and 15 phyla were common to both groups (**Figure 1**). Bacteroidetes (72–81.5%) and Firmicutes (14.4–12.6%) were most abundant among all microbial communities. Verrucomicrobia was the third abundant phylum in the MNFC group. It was also the most significantly expanded phylum compared with the LNFC group (*p* < 0.05). WS3 was only detected in the LNFC group, whereas Elusimicrobia, Armatimonadetes, Gemmatimonadetes, Cyanobacteria, Nitrospira, and Planctomycetes were only detected in the MNFC group. At the genus level, a total of 122 genera were detected in the sequences. Among them, 58 genera were common to both groups (**Figure 1**). The relative abundances of all genera in two groups were shown in Supplementary Table S3. *Prevotella* (38.5–40.7%) was consistently abundant in both groups. Of the genera identified, 49 were only detected in the MNFC group, whereas 14 were only detected in the LNFC group. Non-metric multidimensional scaling (NMDS) plot (Supplementary Figure S1) and the analysis of similarities (ANOSIM) (*p* < 0.05) revealed the divergence of the community structure in the MNFC and LNFC groups.

### Diversity and Richness of Microbial Communities

**FIGURE 2:**
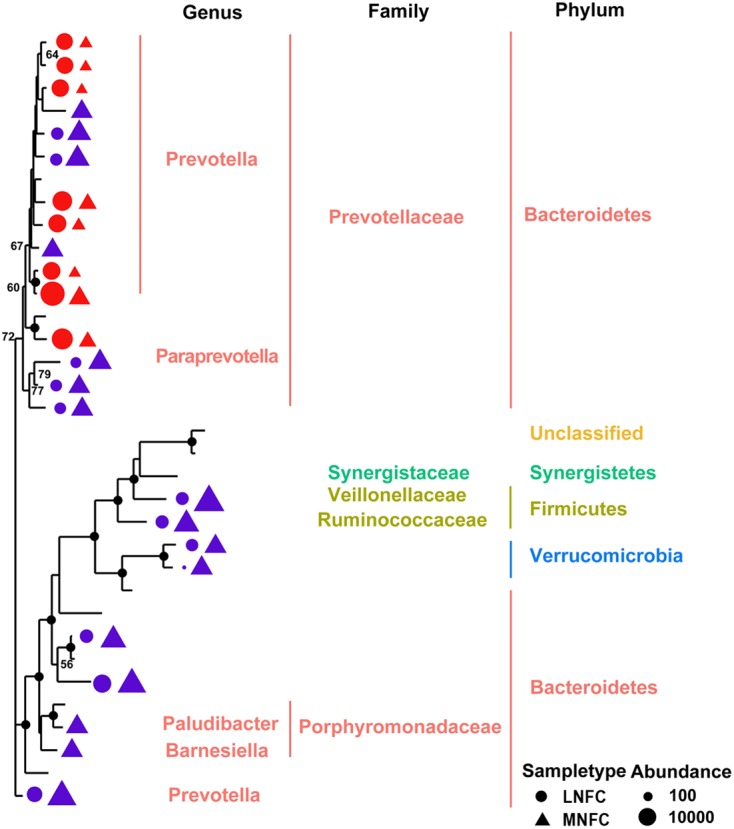
**Maximum likelihood tree of 34 detectable OTUs (relative abundance > 1% for a given sample).** The complete 16S rRNA gene sequences of the corresponding species in the RDP database were used to construct the tree. Triangle indicates the OTUs in the MNFC group, and the circle indicates the OTUs in the LNFC group. Only the OTUs with significant differences (*p* < 0.05) in relative abundance are shown behind the branches. The size of the symbol indicates the relative abundance of OTUs. Blue indicates a significant expansion (*p* < 0.05) of the relative abundance of the OTU after a 31% NFC diet, and red indicates a significant reduction (*p* < 0.05) in the relative abundance of the OTU after a 31% NFC diet. The bootstrap values are shown on the tree. The solid black circles at the nodes stand for the bootstrap value of 100.

The Shannon and Simpson indices revealed that the diversity of microbial communities in the MNFC group was significantly higher than that in the LNFC group (*p* < 0.05) (Supplementary Figure S2). The rarefaction curves showed that the richness of the commensal communities in the MNFC group was significantly higher than that in LNFC group (Supplementary Figure S2). To investigate the richness and diversity of the major phylogenetic clades, a maximum likelihood (ML) tree was constructed from the 16S rRNA sequences of 34 detectable OTUs (the relative abundance > 1%) (**Figure 2**). On the tree, the majority of the significantly expanded or newly detected OTUs in the MNFC group belonged to the families Prevotellacea, Porphyromonadaceae, Ruminococcaceae, Synergistaceae, Veillonellaceae, and unclassified family of Verrucomicrobia. On the contrary, the significantly reduced OTUs unexceptionally belonged to the family Prevotellacea.

### Biomarker Genera within the Microbial Community

**FIGURE 3:**
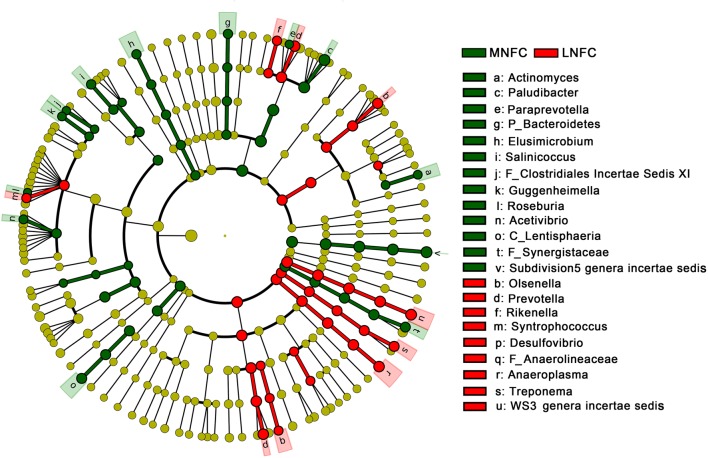
**LEfSe analysis indicating the biomarker genera in the microbial community of MNFC and LNFC groups**.

LEfSe combined rank sum tests and taxonomic information to find the biomarker species with the greatest impact on the structure of the community. In our study, 13 genera were selected as biomarkers for the MNFC group, and nine genera were selected as biomarkers for the LNFC group. The list of the biomarker genera was shown in **Figure 3**.

### Expressions of Genes Related to TLR Signaling and Cytokines

**FIGURE 4:**
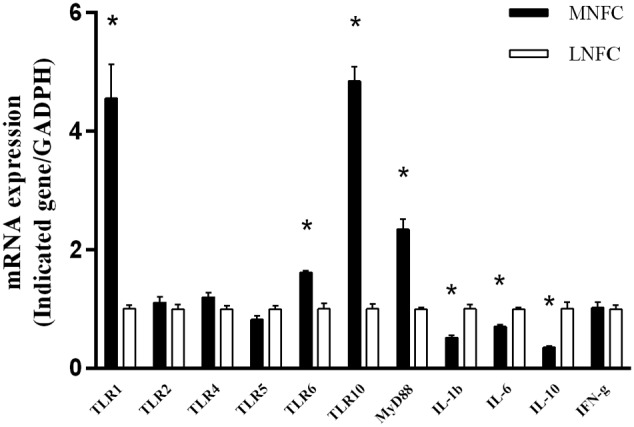
**Comparisons of the expressions of genes related to TLR signaling and pre-inflammatory cytokines.** All analyses were performed in triplicate. “^∗^” indicates a *p*-value < 0.05 in the *t*-test.

After a 4-week period of MNFC feeding, *TLR1*, *TLR6*, *TLR10*, and *MyD88* were significantly increased (*p* < 0.05), whereas interleukin-1beta (*IL-1β*), *IL-6*, and *IL-10* were significantly decreased (*p* < 0.05). In addition, *TLR2*, *TLR4* and interferon-gamma (*IFN-γ*) exhibited no significant changes (*p* > 0.05) (**Figure 4**).

### Correlation between the Differentially Expressed TLRs and Bacterial Biomarkers

**FIGURE 5:**
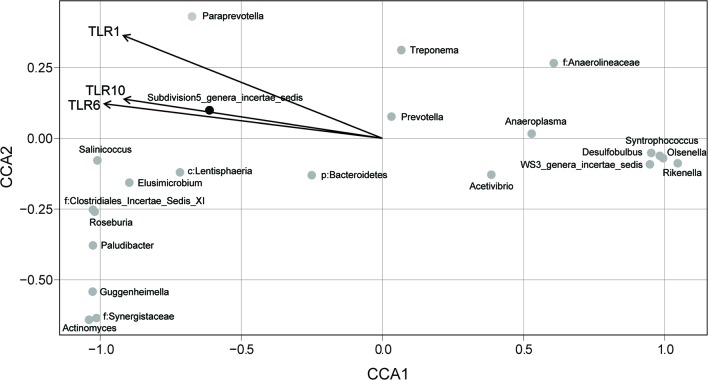
**Constrained correspondence analysis revealing the correlations between the abundance of the microbial biomarkers and the expression of the significantly different TLRs (*p* < 0.05 in *t*-test)**.

Constrained correspondence analysis showed that the expression of *TLR10* was most highly correlated with the relative abundance of unclassified commensals in Verrucomicrobia subdivision 5 (**Figure 5**). No significant correlations were found between the expressions of the remaining *TLRs* and the abundances of biomarker species.

### Dynamics of SCFAs Concentrations and pH in Rumen

**FIGURE 6:**
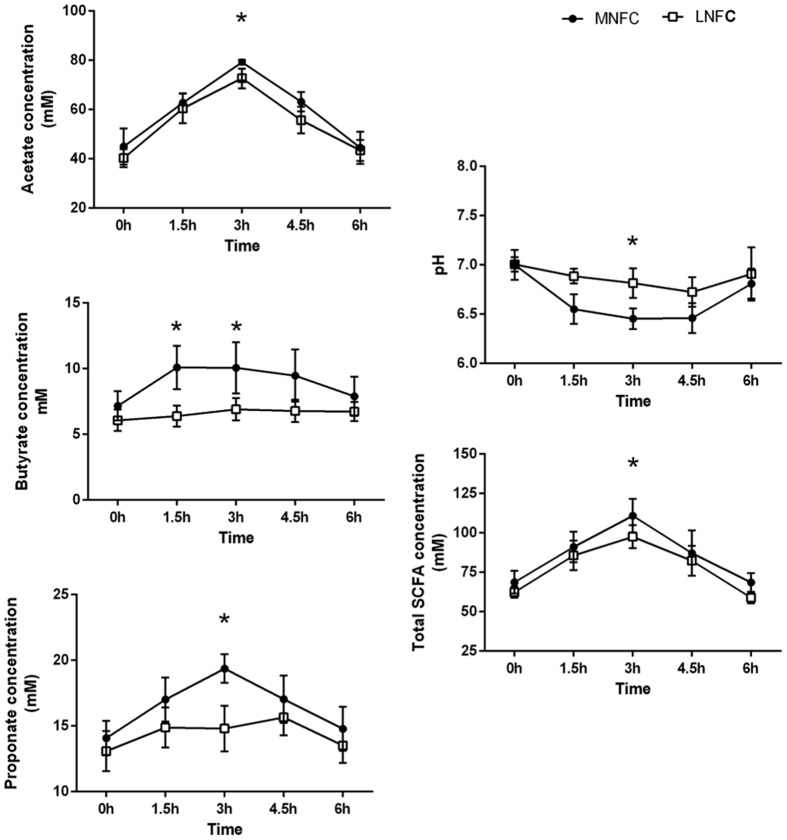
**Effect of diet switch on the concentrations of short-chain fatty acids (SCFAs; acetate, propionate, and butyrate), their molar proportions, total SCFA concentration, and the pH in the ruminal fluid of goats.** 0 indicates the sampling time just before matinal feeding, and other numbers indicate the sampling time after matinal feeding. “^∗^” indicates a *p*-value < 0.05 in the *t*- test.

**Figure 6** shows in MNFC group the concentrations of total SCFA (TSCFA), acetate, propionate and pH significantly increased at 3 h after feeding (*p* < 0.05) respectively, while the concentration of butyrate significantly increased at 1.5 h after feeding (*p* < 0.05) in comparison with LNFC group.

### Expressions of Genes Related to Epithelial SCFA Absorption

**FIGURE 7:**
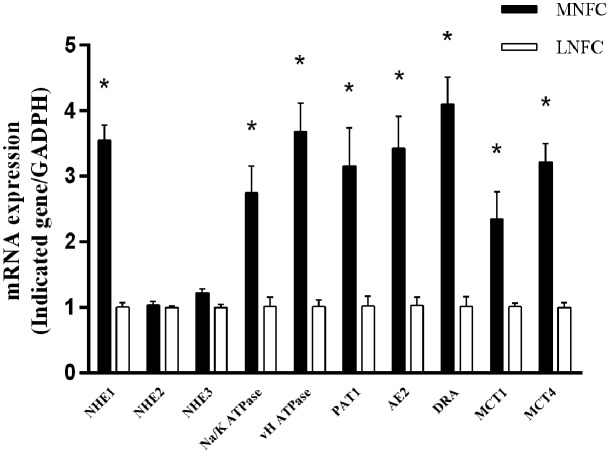
**Comparisons of the expressions of genes related to SCFA absorption of ruminal epithelium.** All analyses were performed in triplicate. “^∗^” indicates a *p*-value < 0.05 in the *t*-test.

With regard to the epithelial genes related to SCFA absorption, e.g., *NHE1*, *NHE3*, sodium-potassium adenosine triphosphatase (*Na^+^/K^+^ ATPase*), vacuolar-type proton adenosine triphosphatase (*vH^+^ ATPase*), putative anion transporter 1 (*PAT1*) anion exchanger 2 (*AE2*), downregulated in adenoma (*DRA*), monocarboxylate transporter 1 (*MCT1*), and monocarboxylate transporter 4 (*MCT4*) were significantly increased (*p* < 0.05), whereas *NHE2* showed no significant change (*p* > 0.05) (**Figure 7**).

## Discussion

Our results demonstrated that the diversity and richness of the microbial community was increased in the MNFC group. Phylogenetic analysis of the detectable OTUs revealed the expansion occurred in all major lineages. Ecological and evolutionary theory suggests that the more diverse communities make a greater contribution to ecosystem functioning. Bell et al. (2005) has proposed that, if each species uses a slightly different nutrient resource and occupies a highly specific niche in the community, then the more diverse microbiota should be able to capture energy, and resistance to invading pathogens more efficient. According to this principle, the MNFC diet should benefit rumen immunity and metabolism, since a more highly diversified ruminal microbiota was observed in the MNFC group. In the present study, the Verrucomicrobia was most significantly expanded after a 4-week period of MNFC feeding. However, this was not detected in the rumen microbiota of goats suffered with diet-induced subacute ruminal acidosis (SARA) (Mao et al., 2016). Previous studies have shown that the bacteria belonging to the Verrucomicrobia play important roles in the acquiring of immune tolerance in mice gut (Derrien et al., 2011). A decrease or absence of these species in the mammalian gut was associated with a decline of host immunity (Png et al., 2010; Swidsinski et al., 2011). Notably, our LEfSe analysis revealed that opportunistic pathogens, namely *Rikenella* (Alkadhi et al., 2014), *Anaeroplasma* (Kalshingi et al., 2015), and *Olsenella* (Vieira Colombo et al., 2016) were significantly shrunk in MNFC group, which might reduce the probability of inflammation in the rumen epithelium as well as ruminant animals. In addition, the concentration of ruminal butyrate was significantly increased in MNFC group. The study of Gantois et al. (2006) has shown that butyrate is able to suppress the expression of virulence genes in bacteria. Thus, the results of our study taken together indicate that an MNFC diet makes commercials bolster their resistance to pathogens. This is, obviously, beneficial to the immunity and health of the host.

Our study has also demonstrated that, in the rumen epithelium, the upregulated expressions of *TLR 1*, *6*, *10*, and *MyD88* expressions were associated with the downregulated expressions of *IL-1β*, *6*, and *10* after a 4-week period of MNFC feeding. Previous studies have shown that, upregulated expressions of pro- and anti- inflammatory cytokines were associated with the unhealthy changes of animals, such as pathogen/virus infection and SARA (Chang et al., 2015; Zhang et al., 2015). In such unhealthy state, pro-inflammatory cytokines enhanced the attack of the immune cells to the pathogens by activating inflammatory responses, and the anti-inflammatory cytokines inhibited immune hurt to the tissue by suppressing inflammatory responses. Accordingly, the synergic decreases of pro-inflammatory cytokines *IL-1β* and *6* and anti-inflammatory cytokine *IL-10* in MNFC group indicated the enhanced tolerance of epithelium to the rumen microbiota.

The study of Oosting et al. (2014) has revealed that antagonistic antibody blocking or siRNA silencing of *TLR10* results in the enhanced production of *IL-1β* and *6* in cultures of human peripheral blood mononuclear cells (PBMCs). Our *in vivo* findings agree with the study of Oosting et al. (2014) by indicating an inhibitory property of *TLR10* signaling on the expression of pre-inflammatory cytokines in the rumen epithelium. In humans, *TLR10* is suggested to exert its function through the formation of heterodimers with *TLR2* (Guan et al., 2010; Oosting et al., 2014; Stappers et al., 2015; Ammerdorffer et al., 2016). However, the upregulation of *TLR10* expression was not associated with any significant change of *TLR2* in our study. By searching for the locations of *TLRs* in the goat genome annotation release 101 in NCBI (Dong et al., 2013), we noted that *TLR10* is located on chromosome 6, together with *TLR1* and *TLR6*, whereas *TLR2* is located on chromosome 17, independently. Moreover, Opsal et al. (2006) have observed the synergy in the expression of *TLR1* and *TLR6* in cattle rumen. We therefore speculate that the inhibitory effect of *TLR10* signaling needs both ligations of the *TLR1/TLR10* complex and of the *TLR6/TLR10* complex in ruminal epithelium. However, more evidence is required from the further studies.

Our CCA revealed that the abundance of the commensal bacterium in Verrucomicrobia subdivision 5 was highly related to the expression of *TLR10*. Previous studies have found that *Akkermansia muciniphila*, the cultured species of Verrucomicrobia, plays an important role in maintaining gut homeostasis (Png et al., 2010; Swidsinski et al., 2011; Hansen et al., 2012; Rajilic-Stojanovic, 2013). This species has also been suggested to be a co-evolutionary bacterium with the mammalian gut (Belzer and de Vos, 2012). An i*n vivo* study in gnotobiotic mice has shown that *A. muciniphila* modulates the expression of pathways related to the immune tolerance of the gut epithelium (Derrien et al., 2011). Thus, we speculated that, in the present study, the suppression of the expressions of pro-inflammatory cytokines might have been caused by the species in Verrucomicrobia subdivision 5 through the activation of *TLR10* signaling.

Higher ruminal concentrations of SCFAs were observed in the MNFC group. This indicated that commensals, induced by the NFC-rich diet, contributed to the increase of SCFAs in the rumen fluid. Previous studies have shown that the upregulated expressions of SCFA^-^/HCO_3_^-^ exchangers *PAT-1*, *AE-2*, and *DRA* were associated with the enhanced uptake of luminal SCFA into the rumen epithelium (Gabel et al., 1991; Kramer et al., 1996; Aschenbach et al., 2009). The upregulated expressions of SCFA- absorption -related transporters *NHE1* and *3*, *Na^+^/K^+^ ATPase*, and *vH^+^ ATPase* contributed to maintain the homeostasis of intracellular pH in a relatively low ruminal pH (Yang et al., 2012). The upregulated expressions of lactate transporters *MCT1* and *4* were associated with the increased transportation of SCFA metabolic products lactate and ketone bodies from the rumen epithelium into the blood for energy supplement (Aschenbach et al., 2011). Accordingly, these data showed that commensals, induced by the NFC-rich diet, were beneficial to the energy absorption of rumen epithelium

## Conclusion

The simultaneous increase of the diversity and richness in the microbiota community, coupled with the decreases in the expressions of pro-inflammatory and anti-inflammatory cytokines, indicates that the NFC-rich diet promoted the tolerance of epithelium to the rumen microbiota. The increased SCFAs productions and upregulated expressions of genes related to the SCFA absorption indicates that the commensals are promoted by the NFC-rich diet. Altogether, our study indicates that a new balanced state is established following 31% NFC intake, and that it is characterized by the facilitated transport of ruminal SCFA into the blood for animal growth. These results provide novel sights into the sustainable development of livestock production through dietary intervention and the development of the next generation of probiotics with the ability to maintain immune homeostasis. They also give hints concerning the recognized ligands of TLR10.

## Author Contributions

HS analyzed data and wrote the paper; ZL designed the research; ZL and ZC performed the experiments; YW and ZS approved the version to be published.

## Conflict of Interest Statement

The authors declare that the research was conducted in the absence of any commercial or financial relationships that could be construed as a potential conflict of interest.
