# Supplementary material for: Rapid Fermentable Substance Modulates Interactions between Ruminal Commensals and Toll-Like Receptors in Promotion of Immune Tolerance of Goat Rumen
Source: Front Microbiol. 2016 Nov 17;7:1812. doi: 10.3389/fmicb.2016.01812 (PMC5112275; doi:10.3389/fmicb.2016.01812)

Fig. S1. Nonmetric multidimensional scaling (NMDS) analysis of Bray-Curtis similarity coefficients based on the relative abundance of OTUs in the corresponding sample.

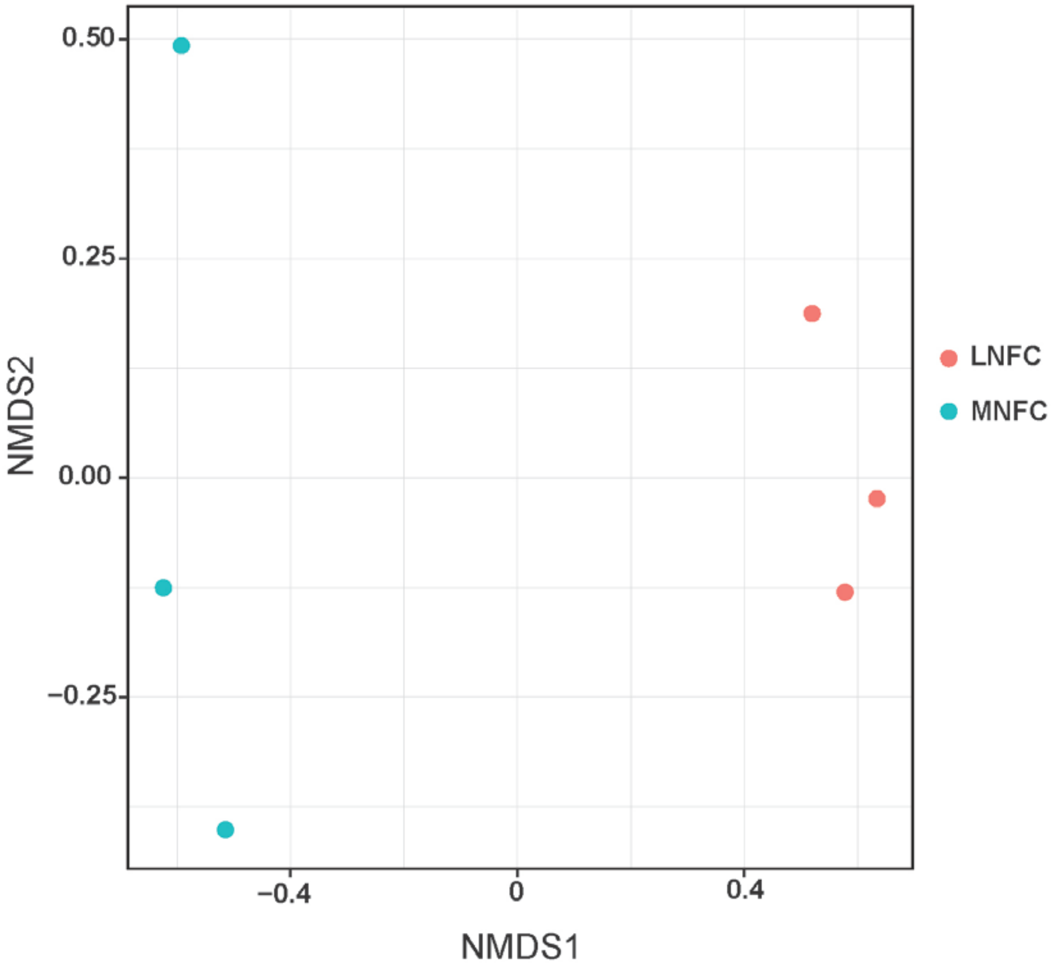

Supplement: Supplementary file 1 [file Image_1.PDF]
