# Supplementary material for: Rapid Fermentable Substance Modulates Interactions between Ruminal Commensals and Toll-Like Receptors in Promotion of Immune Tolerance of Goat Rumen
Source: Front Microbiol. 2016 Nov 17;7:1812. doi: 10.3389/fmicb.2016.01812 (PMC5112275; doi:10.3389/fmicb.2016.01812)

Fig.S2 (A) Community diversity estimated by using Shannon and Simpson indices. (B) Community richness estimated by using rarefaction curves.

(A)

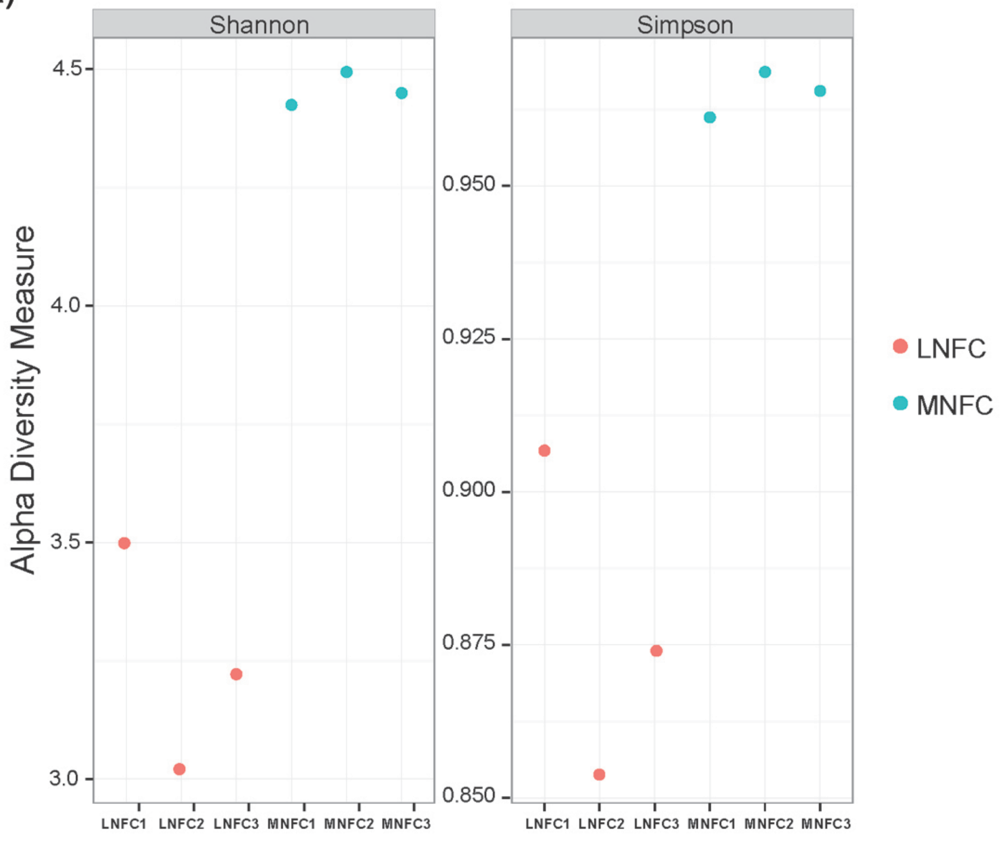

(B)

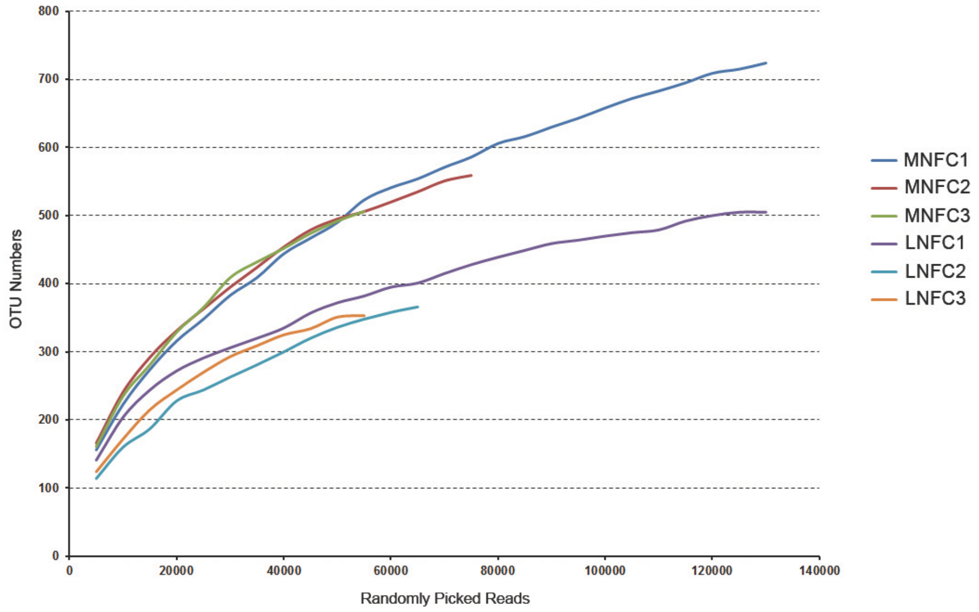

Supplement: Supplementary file 2 [file Image_2.PDF]
