# Supplementary material for: Rapid Fermentable Substance Modulates Interactions between Ruminal Commensals and Toll-Like Receptors in Promotion of Immune Tolerance of Goat Rumen
Source: Front Microbiol. 2016 Nov 17;7:1812. doi: 10.3389/fmicb.2016.01812 (PMC5112275; doi:10.3389/fmicb.2016.01812)
Supplement: Supplementary file 3 [file Table_1.PDF]

Table S1. Dietary compositions used in this study

Ingredients	MNFC	LNFC
Guinea grass	65	90
Corn	25	0
Soya bean meal	8	8
Additive	2	2

The additive was composed of calcium phosphate, limestone, trace mineral salt, and vitamin premix (vitamins A, D, and E).
