# Supplementary material for: Rapid Fermentable Substance Modulates Interactions between Ruminal Commensals and Toll-Like Receptors in Promotion of Immune Tolerance of Goat Rumen
Source: Front Microbiol. 2016 Nov 17;7:1812. doi: 10.3389/fmicb.2016.01812 (PMC5112275; doi:10.3389/fmicb.2016.01812)
Supplement: Supplementary file 4 [file Table_2.pdf]

Table S2 Quantitative PCR primers used in this study

Genes	Reference/Accession Nr.	Forward (5'>3')	Reverse (5'>3')
GAPDH	Yan *et al.*, 2014	TTGTCTCCTGCGACTTCA	CCACCACCCTGTTACTGTT
TLR1	NM_001285605.1	ACAATCCATTCCAATGTTCC	ACAATGGTGACAATCAGCAG
TLR2	XM_013970466.1	CTGTGTGCGTCTTCCTCAGA	TCAGGGAGCAGAGTAACCAGA
TLR4	NM_001285574.1	GGTTTCCACAAAAGCCGTAA	AGGACGATGAAGATGATGCC
TLR5	NM_001285699.1	ACCTGGGTGGAAGTCAGATA	GGTTGAGGGAAAAATCAATG
TLR6	NM_001285540.1	AGGCCAAGTATCAAGAGACG	AGAGGACAGTCACAGCAACA
TLR10	NM_001285541.1	TTGCATGATGGAATCAAAAC	AACCAATTGGAAGATGAGGA
MyD88	XM_013973392.1	ACAAGCCAATGAAGAAAGAG	GAGGCGAGTCCAGAACC
IFN-γ	NM_001285682.1	TGATTCAAATTCCGGTGGAT	GCAGGCAGGAGAACCATTAC
IL-1ß	XM_013967700.1	CATGTGTGCTGAAGGCTCTC	AGTGTCGGCGTATCACCTTT
IL-6	NM_001285640.1	CCAATCTGGGTTCAATCAGG	ACCCACTCGTTTGAGGACTG
IL-10	XM_005690416.2	TTAAGGGTTACCTGGGTTGC	CCCTCTCTTGGAGCATATTGA
NHE1	Yan *et al.*, 2014	CCTCTACAGCTACATGGCCTAC	GGGAGATGTTGGCTTCCA
NHE2	Yan *et al.*, 2014	TTGGAGAGTCCCTGCTGAAC	GGCCGTGATGTAGGACAAAT
NHE3	Yan *et al.*, 2014	AGCTACGTGGCCGAGGG	AGACAGAGGCCTCCACGGT

Genes	Reference/Accession Nr.	Forward (5'>3')	Reverse (5'>3')
Na^+/K^+ ATPase	Yan *et al.*, 2014	TGAGCATCCCAGTGTTGT	CCTTGTCCAGATACTTCCT
vH ATPase	Yan *et al.*, 2014	TTTTATTGAACAAGAAGCCAATGA	GATTCATCAAATTGGACATCTGAA
PAT1	Yan *et al.*, 2014	CCTTGAGGCACGGCTAC	GCACCAGACTCCGAGACATA
AE2	Yan *et al.*, 2014	AGCAGCAACAACCTGGAGT	GGTGAAACGGGAGACGAA
DRA	Yan *et al.*, 2014	TTTAAAGTCCTAGAGTCCGTA	CGCTGATTTATTTCTTTAACCAC
MCT1	Yan *et al.*, 2014	ACCAGTTTTAGGTCGTCTCA	GGCTTCTCAGCAACATCTACA
MCT4	Yan *et al.*, 2014	GTTTGGGATAGGCTACAGTGACACA	GCAGCCAAAGCGATTCACA
