# Supplementary material for: Rapid Fermentable Substance Modulates Interactions between Ruminal Commensals and Toll-Like Receptors in Promotion of Immune Tolerance of Goat Rumen
Source: Front Microbiol. 2016 Nov 17;7:1812. doi: 10.3389/fmicb.2016.01812 (PMC5112275; doi:10.3389/fmicb.2016.01812)
Supplement: Supplementary file 5 [file Table_3.pdf]

Table S3. Rumen microbial compositions of MNFC and LNFC groups (mean value ± SE; n = 3).

Item	Genus	MNFC±SE	LNFC±SE
1	Prevotella	38531±5348	40653±1013
2	p:Bacteroidetes	8629±2112	5412±1241
3	o:Bacteroidales	8214±414	30450±4418
4	f:Prevotellaceae	5333±929	2446±169
5	Paraprevotella	5238±2073	896±126
6	Subdivision5_genera_IS	4423±458	1086±673
7	f:Lachnospiraceae	3980±256	950±169
8	f:Ruminococcaceae	3341±611	3140±157
9	f:Synergistaceae	3270±2171	42±33
10	f:Porphyromonadaceae	2973±1155	1378±209
11	f:Veillonellaceae	2453±1439	4473±212
12	Unclassified_Bacteria	1808±815	1359±218
13	Paludibacter	1686±767	0
14	Barnesiella	1346±655	260±17
15	o:Clostridiales	1138±262	1589±223
16	Vampirovibrio	927±482	343±45
17	Victivallis	784±204	246±138
18	Treponema	707±463	787±132
19	p:Firmicutes	700±297	652±375
20	Ruminococcus	414±23	152±13
21	Oscillibacter	405±111	6±5
22	c:Lentisphaeria	401±116	69±21
23	Clostridium_IV	340±148	47±7
24	o:Selenomonadales	323±139	21±22
25	Lachnospiracea_IS	310±36	1018±15
26	Butyrivibrio	255±79	176±56
27	TM7_genera_IS	242±57	309±87
28	Synergistes	185±89	25±24
29	Anaerovibrio	118±36	14±6
30	Salinicoccus	112±67	0
31	SR1_genera_IS	92±55	114±48
32	Hallella	91±88	19±20
33	Anaeroplasma	90±28	324±78
34	Acetivibrio	87±28	213±54
35	c:Alphaproteobacteria	86±37	516±154
36	p:Proteobacteria	83±44	83±43
37	Elusimicrobium	74±31	0
38	Anaerovorax	57±29	47±19
39	Succiniclasticum	55±41	16±10
40	Sporobacter	51±10	14±12
41	Schwartzia	45±18	17±3
42	Fibrobacter	43±29	123±30

43	c:Epsilonproteobacteria	37±23	2±4
44	Pseudobutyrivibrio	34±16	21±3
45	Actinomyces	33±21	0
46	Coprococcus	31±14	4±4
47	Guggenheimella	30±22	0
48	Blautia	26±5	0
49	Succinivibrio	25±9	95±41
50	f:Enterobacteriaceae	24±7	18±10
51	Slackia	24±25	4±5
52	o:Actinomycetales	23±7	0
53	f:Coriobacteriaceae	19±17	5±4
54	f:Erysipelotrichaceae	17±7	27±12
55	f:Clostridiales_IS_XI	16±14	0
56	Saccharofermentans	15±4	3±5
57	Nocardioides	15±25	0
58	Petrimonas	14±12	0
59	c:Deltaproteobacteria	12±11	13±5
60	Roseburia	11±5	0
61	Dongia	11±18	0
62	Cerasicoccus	10±10	4±7
63	Howardella	10±9	1±2
64	Virgibacillus	9±8	0
65	Spirochaeta	8±14	1±2
66	Mogibacterium	8±7	0
67	f:Xanthomonadaceae	8±14	0
68	Selenomonas	7±8	1±2
69	Propionibacterium	7±8	0
70	f:Bacillaceae	7±8	0
71	c:Clostridia	6±11	8±8
72	Proteiniphilum	6±6	0
73	Solobacterium	6±6	0
74	Gp1	4±7	0
75	c:Actinobacteria	4±7	0
76	o:Sphingobacteriales	4±6	0
77	Clostridium_XlVa	4±6	0
78	Gemmatimonas	4±7	0
79	Caulobacter	4±8	0
80	f:Comamonadaceae	4±8	0
81	f:Oxalobacteraceae	4±8	0
82	Yaniella	2±3	8±1
83	Streptococcus	2±3	5±5
84	o:Acidimicrobiales	2±3	1±2
85	Desulfobulbus	2±3	1±2
86	Gp3	2±3	0

87	Gp4	2±3	0
88	Actinospica	2±3	0
89	Atopobium	2±3	0
90	Armatimonadetes_gp2	2±3	0
91	Armatimonadetes_gp4	2±3	0
92	Flavisolibacter	2±3	0
93	o:Ktedonobacterales	2±3	0
94	Sphaerobacter	2±3	0
95	p:Chloroflexi	2±3	0
96	Chlorophyta	2±3	0
97	Bacillus	2±3	0
98	Tissierella	2±3	0
99	Acetanaerobacterium	2±3	0
100	Nitrospira	2±3	0
101	Gemmata	2±3	0
102	o:Rhodospirillales	2±3	0
103	Massilia	2±3	0
104	f:Sutterellaceae	2±3	0
105	Ruminobacter	2±3	0
106	Moraxella	2±3	0
107	Pseudomonas	2±3	0
108	Desulfovibrio	0	130±18
109	Olsenella	0	70±23
110	f:Anaerolineaceae	0	31±28
111	Syntrophococcus	0	16±2
112	Rikenella	0	15±6
113	Escherichia/Shigella	0	6±5
114	Oribacterium	0	5±9
115	Bilophila	0	5±5
116	Eubacterium	0	4±4
117	f:Desulfovibrionaceae	0	4±4
118	WS3_genera_IS	0	4±5
119	Rothia	0	1±2
120	Enterorhabdus	0	1±2
121	Pusillimonas	0	1±2

Counts were normalized to the relative abundance of each sample and showed here in a scale of 1/100,000; genera were sorted based on their relative abundance in the rumen microbial community in a descending order; f = Family; o = Order; c = Class; p = Phylum.
